# Unravelling the Therapeutic Potential of Antibiotics in Hypoxia in a Breast Cancer MCF-7 Cell Line Model

**DOI:** 10.3390/ijms241411540

**Published:** 2023-07-16

**Authors:** Almaz A. Akhunzianov, Alfiya I. Nesterova, Sjoerd Wanrooij, Yulia V. Filina, Albert A. Rizvanov, Regina R. Miftakhova

**Affiliations:** 1 Institute of Fundamental Medicine and Biology, Kazan Federal University, 420008 Kazan, Russia; aaahunzyanov@kpfu.ru (A.A.A.); alfikhasanova@kpfu.ru (A.I.N.);; 2 Republican Clinical Oncology Dispensary Named after Prof. M.Z. Sigal, 420029 Kazan, Russia; 3 Department of Medical Biochemistry and Biophysics, Faculty of Medicine, Umeå University, 907 36 Umeå, Sweden; sjoerd.wanrooij@umu.se

**Keywords:** antibiotics, breast cancer, cancer stem cells, hypoxia, mitochondria

## Abstract

Antibiotics inhibit breast cancer stem cells (CSCs) by suppressing mitochondrial biogenesis. However, the effectiveness of antibiotics in clinical settings is inconsistent. This inconsistency raises the question of whether the tumor microenvironment, particularly hypoxia, plays a role in the response to antibiotics. Therefore, the goal of this study was to evaluate the effectiveness of five commonly used antibiotics for inhibiting CSCs under hypoxia using an MCF-7 cell line model. We assessed the number of CSCs through the mammosphere formation assay and aldehyde dehydrogenase (ALDH)-bright cell count. Additionally, we examined the impact of antibiotics on the mitochondrial stress response and membrane potential. Furthermore, we analyzed the levels of proteins associated with therapeutic resistance. There was no significant difference in the number of CSCs between cells cultured under normoxic and hypoxic conditions. However, hypoxia did affect the rate of CSC inhibition by antibiotics. Specifically, azithromycin was unable to inhibit sphere formation in hypoxia. Erythromycin and doxycycline did not reduce the ratio of ALDH-bright cells, despite decreasing the number of mammospheres. Furthermore, treatment with chloramphenicol, doxycycline, and tetracycline led to the overexpression of the breast cancer resistance protein. Our findings suggest that hypoxia may weaken the inhibitory effects of antibiotics on the breast cancer model.

## 1. Introduction

At an American Association for Cancer Research (AACR) Workshop in 2006, Cancer Stem Cells (CSCs) were defined as cells within a tumor possessing the ability to self-renew and generate heterogeneous lineages of cancer cells [1]. Accumulating evidence suggests that CSCs are responsible for tumor metastasis [1], evasion of antitumor immune responses [2], and the establishment of protective niches that sustain both their proliferation and quiescence [3].

CSCs, like normal stem cells, prefer glycolysis as an energy production pathway over oxidative phosphorylation [4]. Furthermore, stem cells exhibit fewer mitochondria and suppressed mitochondrial biogenesis compared to differentiated cells [5]. This characteristic is crucial for stem cells to maintain low levels of reactive oxygen species (ROS), which are necessary to preserve genome integrity and a quiescent state. CSCs utilize glucose and require a replete supply of this energy source [6]. However, several studies have demonstrated that CSCs are even more reliant on glycolysis for energy production than bulk tumor cells. In other words, CSCs exhibit higher levels of adenosine triphosphate (ATP) production, glycolytic enzyme expression, and glucose uptake, along with a simultaneous decrease in mitochondrial number and a reduction in oxidative phosphorylation rates [7,8]. Conversely, our current understanding of this topic is still limited and contradictory, as some studies have reported that CSCs are less glycolytic and exhibit an enhanced rate of oxidative phosphorylation, active mitochondrial metabolism, and an increased number of mitochondria [9]. In such cases, CSCs likely redirect their metabolism from oxidative phosphorylation to the glycolytic pathway under hypoxic conditions [10]. Recently, a study addressed and clarified this discrepancy, revealing that a metabolic shift from glycolysis to an oxidative mitochondrial phenotype occurs in CSCs during the transition from anchorage-dependent to anchorage-independent growth [11]. In summary, mitochondrial biogenesis plays a critical role in maintaining the CSC phenotype.

According to the symbiogenesis theory of mitochondrial evolution, which is supported by the high homology between mitochondrial and prokaryotic proteins, mitochondria originally evolved from prokaryotic cells engulfed by eukaryotes. Bacterial and mitochondrial ribosomes share structural similarities. The 39S large mitoribosomal subunit contains nearly 48 proteins, most of which are homologs of bacterial ribosomal proteins [12]. Similarly, the 28S small mitoribosomal subunit consists of approximately 29 proteins, 14 of which have homologs in prokaryotic ribosomes. Consequently, many classes of bactericidal antibiotics may target mitochondrial biogenesis as a side effect. For instance, prolonged treatment with quinolones, aminoglycosides, and β-lactams can lead to overproduction of ROS and mitochondrial dysfunction in mammalian cells. Tetracyclines bind to the small subunit of the ribosome in mitochondria and disrupt mitochondrial translation, while erythromycins inhibit the translation of oxidative phosphorylation proteins by binding to the large subunit of mitoribosomes. Therefore, translation-inhibiting antibiotics should be used carefully in patients with defects in mitochondrial translation machinery [13]. Nevertheless, targeting mitochondrial metabolism with antibiotics appears to be a promising strategy for eliminating the CSC population. Several in vivo and in vitro studies conducted in the last decade using United States Food and Drug Administration (FDA) approved antibiotics have demonstrated their potential to inhibit CSC subpopulations [14,15]. R. Lamb et al. identified a strict dependence on the mitochondrial biogenesis of CSCs as a common phenotypic weak point across multiple tumor types. They propose a therapeutic strategy for eradicating CSCs based on inhibiting mitochondrial biogenesis. According to the research, four different FDA-approved antibiotics (azithromycin, doxycycline, tigecycline, chloramphenicol) can be employed to eliminate CSCs in twelve different cancer cell lines representing eight distinct tumor types. Importantly, these drugs do not exhibit toxicity towards normal cells [16]. Recent clinical trials with azithromycin and doxycycline have shown positive therapeutic effects in cancer patients, although the antibiotics were used to treat cancer-associated infections rather than targeting the CSC population [17,18,19]. Azithromycin was reported to enhance the favorable results of paclitaxel and cisplatin treatment in patients with advanced non-small-cell lung cancer [19]. The one-year survival rate in the group receiving azithromycin combined with chemotherapy was 75.0%, compared to 45.0% in the chemotherapy-only group.

Hypoxia is a significant phenomenon that influences tumor cells’ biology, remodels their metabolism, and contributes to tumor progression [20]. Hypoxia-inducible factors (HIFs) are key elements that orchestrate the cellular response to low oxygen levels in both healthy and tumor cells [21]. HIF-regulated target genes include those involved in angiogenesis, proliferation, apoptosis, survival, and adaptation to insufficient oxygen levels, such as glycolytic enzymes [22,23]. The response to hypoxia regulated by HIFs appears to be gradual and dependent on the available oxygen level [24]. Clinical data demonstrate that overexpression of HIF, either due to genetic mutations or exposure to hypoxia, is associated with increased mortality in patients [25]. For instance, high levels of HIF1α or HIF2α expression in tumor biopsies from breast, pancreatic, or lung cancer patients correlate with an increased incidence of metastasis and mortality [26]. HIF plays a crucial role in protecting cancer cells from hypoxic stress by shifting cell metabolism from oxidative phosphorylation in mitochondria to cytoplasmic glycolysis [10].

The objective of our research was to evaluate the effectiveness of five commonly used antibiotics in inhibiting the subpopulation of breast cancer stem cells of Michigan Cancer Foundation-7 (MCF-7) cells under both normoxic and hypoxic conditions. We employed a tumor sphere formation assay and measured aldehyde dehydrogenase (ALDH) activity to assess the inhibitory activity of bactericidal antibiotics on the CSC population.

## 2. Results

### 2.1. Antibiotics Do Not Affect Proliferation of Cancer Cells in Anchorage-Dependent Culture

**Figure 1:**
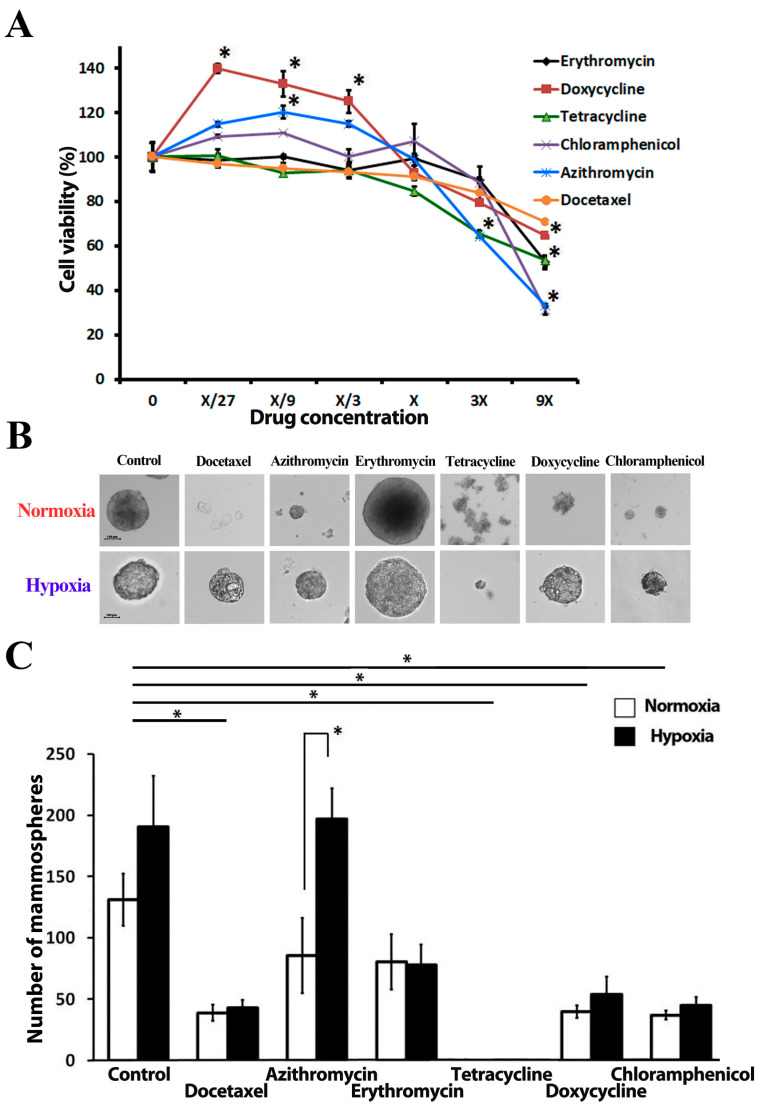
The effect of tested antibiotics on MCF-7 breast cancer cell proliferation and mammosphere formation: (**A**) MTT cell proliferation assay, X-concentration of antibiotics used in sphere formation experiments; (**B**) Representative photomicrographs of MCF-7 mammospheres under normoxia and hypoxia; (**C**) Number of mammospheres after 2 weeks of incubation under both normoxia (20% O_2_) and hypoxia (4% O_2_); shown are the means of 5 independent experiments using MCF-7 cells of different passages. * = *p* < 0.05.

To find the optimal antibiotic concentrations for further experiments, we tested the cytotoxicity of five antibiotics—azithromycin, chloramphenicol, doxycycline, erythromycin, and tetracycline—on MCF-7 breast cancer cells grown as a conventional anchorage-dependent cell culture monolayer. Six different concentrations were chosen for each antibiotic. The range of concentrations is presented in Table 1. For each compound, we determined the highest concentration that did not inhibit proliferation of the bulk population of cancer cells (Figure 1A: concentration X for each antibiotic); this concentration is presented in Table 1 and has been chosen for all experiments in the study. We have also observed a significant increase in cancer cell proliferation in the presence of low concentrations of doxycycline and azithromycin. It has been previously reported that, under some certain circumstances, drug exposure itself may induce intratumoral or systemic changes, which paradoxically exacerbate cancer cell proliferation and dissemination in patients [27,28]. The highest concentrations of all five antibiotics decreased the proliferation of tumor cells. The possible mechanisms for anti-proliferative effects on cancer cells have been linked to an impairment of mitochondrial protein synthesis [29], cell cycle arrest [30], and induction of apoptosis by caspase-3 activation [31].

### 2.2. Antibiotics Affect Mammosphere Formation under Hypoxia and Normoxia

The tumor sphere formation assay (SFA) is a widely used approach to evaluate the frequency of CSCs in a total cancer cell population [16]. Spheres present dense round structures, formed by a single CSC proliferation and floating freely in sphere culture media, thereby representing an anchorage-independent growth condition of cancer cells. Sphere formation is a property that stem cells or tumor-initiating cells from different lineages share in common. MCF-7 CSCs form mammospheres with diameter ranges of 100–350 µm in a suspension culture within 10–12 days (Figure 1B). The sphere number did not depend on oxygen supply conditions; no significant difference was observed between spheres cultured in normoxia (131.3 ± 21.2) or hypoxia (190.8 ± 41.4) (Figure 1C). We detected a significant difference in the average spheres’ diameter in the control samples cultured in normoxia and hypoxia: 205.6 ± 6.4 µm vs. 143.6 ± 8.9 µm, respectively. Hypoxic conditions inhibit MCF-7 cell proliferation but promote cell migration according to the earlier experiment results [32]. A similar decrease in cell proliferation due to hypoxia is reported for most cell types [33]. A greater number of cells will consequently increase the oxygen demand, thus additionally exacerbating hypoxic stress. Docetaxel was introduced to the study as a positive control compound and caused a significant reduction in sphere number, regardless of the oxygen level in a culture environment (decrease by 69.0 ± 4.2% in normoxia vs. 68.4 ± 10.6% in hypoxia).

Tetracycline at a concentration, which did not inhibit cell proliferation in conventional anchorage-dependent cell culture, led to the complete elimination of spheres in both normoxic and hypoxic conditions. This observation might be explained by the early degradation of spheres. A significant decrease in MCF-7 sphere formation capacity has been detected in the presence of doxycycline (25 µM) and chloramphenicol (250 µM) under both normoxic and hypoxic conditions: 68.24 ± 3.12% and 70.53 ± 6.77% for doxycycline, 67.13 ± 6.09% and 68.45 ± 9.40% for chloramphenicol in normoxic and hypoxic conditions, accordingly (Figure 1C).

Interestingly, we have observed differences in sphere count between hypoxic and normoxic conditions in the presence of azithromycin (Figure 1C): 85.8 ± 33.1 and 197 ± 25.3 in normoxia and hypoxia, respectively. We also noticed that some antibiotics cause constant changes in mammosphere morphology (Supplementary Material, Figure S1). In the presence of erythromycin, spheres become larger when compared with spheres from control wells: 223.6 ± 74.9 µm vs. 134.7 ± 13.7 µm in the control group. Docetaxel also influenced spheres’ shape and morphology, yielding «blastocyst-like» spheres.

### 2.3. Antibiotics Affect ALDH-Bright Cell Number under Hypoxia and Normoxia

**Figure 2:**
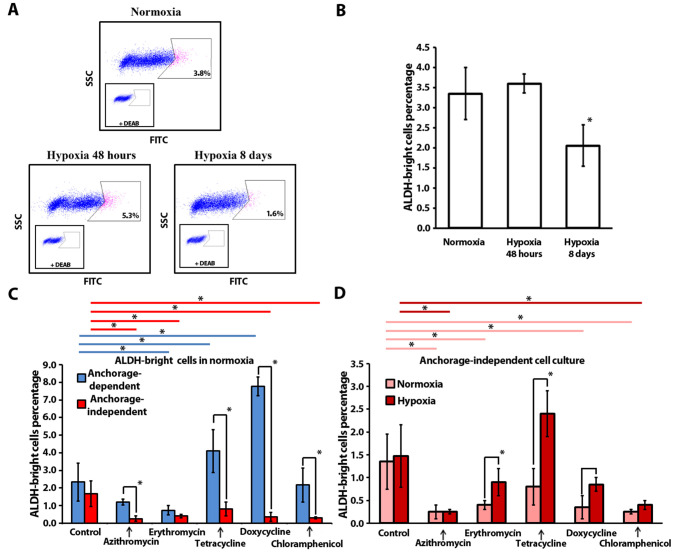
Flow cytometry analysis of ALDH-bright cell ratio in MCF-7 cells: (**A**) Representative flow cytometry plots illustrating the typical gating strategy used to identify the ALDH-bright cells (pink color); (**B**) ALDH-bright cell ratio in a total population of MCF-7 cells in 2D-culture under normoxia (20% O_2_) and after exposure to hypoxia (4% O_2_); (**C**) ALDH-bright cells’ relative percentage after antibiotic treatment in 2D-culture (monolayer culture) and 3D-culture (mammosphere culture) under normoxic conditions, blue lines (**----**) and red lines (**----**) indicate statistically significant differences in monolayer culture and mammosphere culture, respectively (n = 3; *—*p* ≤ 0.05); (**D**) ALDH-bright cell ratio in a population of MCF-7 cells from mammospheres after a 2-week treatment, pink lines (**----**) and red lines (**----**) indicate statistically significant differences in normoxia and hypoxia, respectively (n = 3; *—*p* ≤ 0.05).

Over the past decades, accumulating evidence suggest that high ALDH activity is an important marker of stem and progenitor cells in breast cancer, associated with metastatic ability, high malignancy, and tumor cell proliferation rates [34]. Additionally, ALDH superfamily enzymes are key regulators of molecular pathways, related to differentiation, self-renewal, and drug resistance [35]. Therefore, we questioned how antibiotics influence the ALDH-positive cell population in conventional cell culture (anchorage-dependent cell culture conditions) and in mammosphere cell culture (anchorage-independent culture). Initially, we tested how oxygen levels influence the ALDH-bright cell ratio in an MCF-7 population in conventional culture under hypoxic and normoxic conditions (Figure 2A,B). The ALDH-bright cell percentage is relatively constant under normoxia if the cells are cultured with regular passaging and strict culturing rules. Our data indicate that 3.4 ± 0.7% of MCF-7 cells display high ALDH expression in normoxia. Hypoxic stress (4% O_2_) during the first 48 h did not affect the ALDH-bright cell percentage. Long-term exposure to hypoxia resulted in a significant decrease (*p* ≤ 0.05) in the ALDH-bright cell percentage (2.1 ± 0.5%), observed after eight days of culture with regular cell passaging at hypoxic conditions (Figure 2B).

Furthermore, we cultured MCF-7 cells in anchorage-dependent and independent conditions under normoxia in the presence of antibiotics and analyzed the ALDH-bright cell ratio (Figure 2C). In normoxia no difference was observed in the ALDH-positive cell percentage of the control samples, cultured in anchorage-dependent and independent conditions.

In the anchorage-dependent culture, tetracycline and doxycycline treatment resulted in a statistically significant increase in the ALDH-bright cell percentage: 4.1 ± 1.2% and 7.8 ± 0.5%, accordingly, as compared to the control culture (2.3 ± 1.1%). This increase might be due to the elimination of the bulk cancer cells via elevating the ROS level, thus leading to the enrichment of ALDH-positive cells [36]. A statistically significant decrease in the ALDH-positive cell percentage was observed in the presence of erythromycin: 0.7 ± 0.3% vs. 2.3 ± 1.1%.

Interestingly, in normoxia, in anchorage-independent culture, azithromycin (0.3 ± 0.2%), erythromycin (0.4 ± 0.1%), doxycycline (0.4 ± 0.3%) and chloramphenicol (0.3 ± 0.1%) significantly reduced the ALDH-bright cell ratio, as compared to the control samples (1.7 ± 0.7%).

Furthermore, we compared the ALDH-positive cell ratio for each compound in anchorage-dependent and anchorage-independent culture conditions in normoxia (Figure 2C). With four out of five tested antibiotics we observed a statistically significant decrease in the ALDH-bright cell percentage in spheres, as compared to conventional cell culture: azithromycin (4-fold change), tetracycline (5-fold change), doxycycline (19-fold change) and chloramphenicol (7-fold change). Thus, in normoxia, doxycycline both inhibits sphere formation and reduces the ALDH-bright cell population in the sphere culture. Among all antibiotics, only erythromycin induced a reduction in the ALDH-bright cell percentage in both cell culture models. In conclusion, the antibiotics’ effect on the ALDH-positive cell ratio depends on the culture conditions. In general, four tested compounds (azithromycin, erythromycin, doxycycline, and chloramphenicol) reduced the ALDH-bright cell ratio in spheres in anchorage-independent conditions.

In addition, we analyzed the ALDH-positive ratio of cells treated with antibiotics in anchorage-independent conditions under normoxia and hypoxia (Figure 2D). In the control culture, hypoxia did not affect the ALDH-bright cell ratio in the breast cancer MCF-7 cell line model (1.4 ± 06% in normoxia vs. 1.5 ± 0.7% in hypoxia).

In hypoxia, in anchorage-independent culture, there was a significant decrease in the ratio of ALDH-bright cells in the presence of azithromycin (0.3 ± 0.1%) and chloramphenicol (0.4 ± 0.1%), as compared to the control samples (1.5 ± 0.7%) (Figure 2D). It is important to highlight that doxycycline reduced both the sphere number and the ALDH-bright cell ratio in normoxia but inhibited solely the sphere formation in hypoxia without any impact on the ALDH-positive cell ratio in the anchorage-independent MCF-7 cell population (Figure 2C,D).

Furthermore, we compared the ALDH-bright cell ratio in spheres in relation to the oxygen levels. We observed a significant increase in the ALDH-bright cell ratio between cells cultured under hypoxia in the presence of erythromycin (2-fold increase), tetracycline (3-fold increase), and doxycycline (2-fold increase) as compared to normoxia.

To summarize, four out of five tested antibiotics caused a decrease in ALDH-bright cells in an anchorage-independent normoxic environment, while the lowering of oxygen or modification of cell adhesion features induced an antibiotic-specific response in the stem cell ratio.

### 2.4. Doxycycline, Tetracycline, and Chloramphenicol Affect Mitochondrial Membrane Polarization and Inhibit Mitochondrial Metabolism

**Figure 3:**
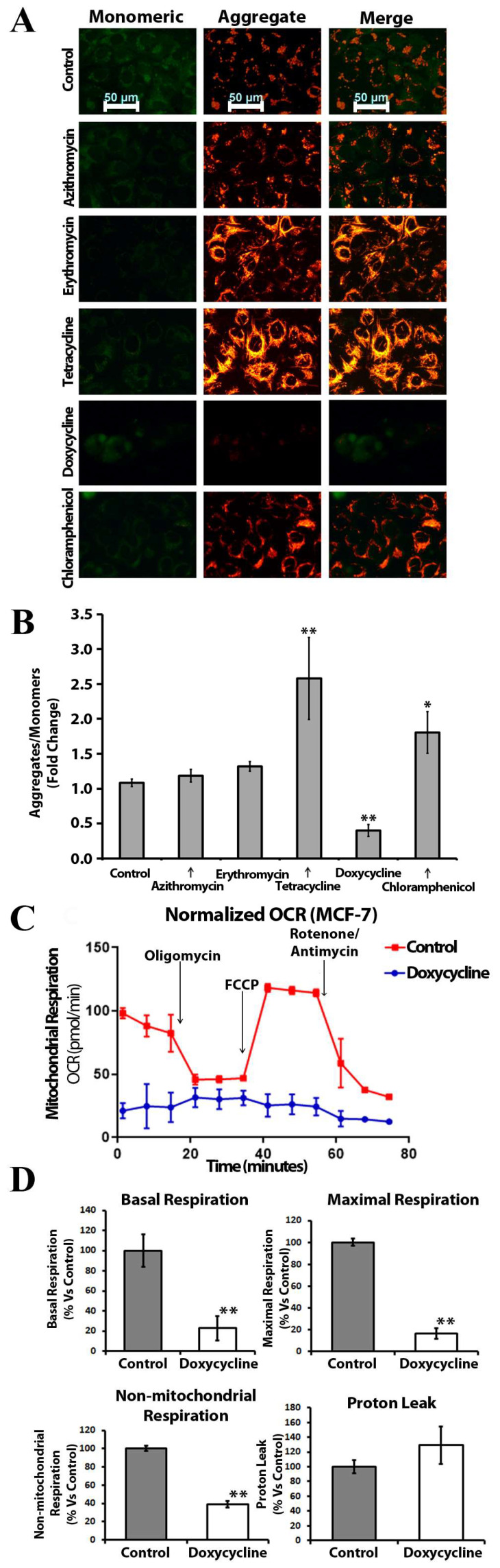
Doxycycline induces mitochondrial dysfunction in MCF-7 cells: (**A**) JC-1 staining of mitochondrial network in MCF-7 cells after exposure to antibiotics (the representative images), shift from red to green fluorescence represents depolarization of mitochondrial membrane potential. Scale bar = 50 μm; (**B**) MCF-7 cells after treatment were subjected to JC-1 staining. The ratio of JC-1 aggregates to monomer was significantly decreased in presence of doxycycline and increased with tetracycline and chloramphenicol. Error bars represent S.E.M. (n = 3; *—*p* ≤ 0.05; **—*p* ≤ 0.01); (**C**) Doxycycline inhibits both mitochondrial and non-mitochondrial respiration in MCF-7 cells; the metabolic profile of MCF-7 cell monolayers treated with doxycycline (25 μM) was assessed using the Seahorse XF-e96 analyzer; representative tracing of metabolic flux; (**D**) Dose-dependent significant reduction in basal respiration, maximal respiration, and non-mitochondrial respiration were observed (n = 3; **—*p* ≤ 0.01).

Depolarization of the mitochondrial membrane is one of the early steps of drug-induced apoptosis [37]. We tested the influence of the five antibiotics on mitochondrial membrane potential in MCF-7 cells (Figure 3A) and human skin fibroblasts (HSF) (Supplementary Material Figure S2) with 5,5,6,6′-tetrachloro-1,1′,3,3′ tetraethylbenzimi-dazoylcarbocyanine iodide (JC-1) a cationic dye that accumulates in mitochondria and gives different fluorescence depending on mitochondrial polarization status. This dye exists as a monomer at low concentrations and yields green fluorescence when the mitochondrial membrane potential is low. In contrast, if mitochondrial membrane potential is high, JC-1 accumulation causes a fluorescence emission shift from green to red due to concentration-dependent formation of red fluorescent aggregates.

The ratio of JC-1 aggregates to monomers was significantly higher in the presence of chloramphenicol and tetracycline, indicating an elevated mitochondrial transmembrane potential (Figure 3B). We detected depolarization of the mitochondrial membrane in the presence of doxycycline in MCF-7 cells.

To understand the effects of doxycycline treatment on breast cancer cell metabolism, we performed metabolic flux analysis and measured the oxygen consumption rate (OCR) with the Seahorse XFe96 Analyzer (Seahorse Bioscience, MA, USA). Interestingly, we observed a dramatic reduction in OCR in MCF-7 cells treated with doxycycline as compared to control cells: there was a decrease in both maximal and basal respiration (Figure 3C). Thus, doxycycline inhibits oxidative phosphorylation and impairs mitochondrial function in MCF-7 cells. It is also noteworthy that there was a reduction in non-mitochondrial respiration after exposure to doxycycline (Figure 3D), indicating the suppression of overall metabolism in MCF-7 cells.

### 2.5. Anchorage-Independent Long-Term Culture with Tetracycline, Doxycycline, and Chloramphenicol Leads to an Increase in ABCG2 Protein Level in Normoxia

**Figure 4:**
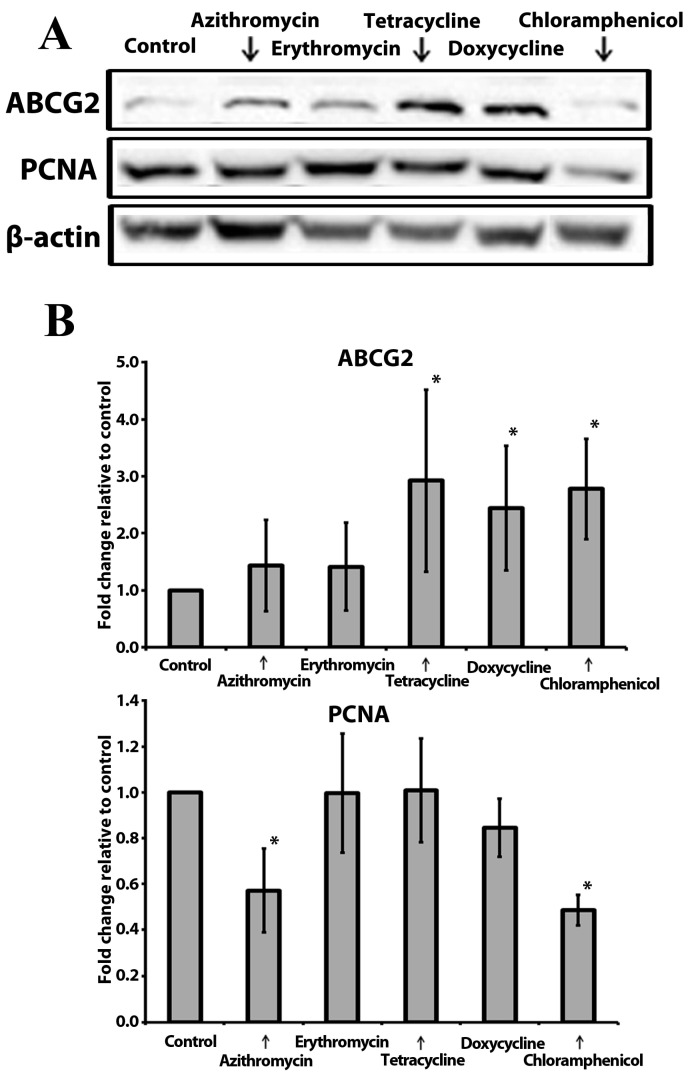
Effect of tested compounds on PCNA and ABCG2 protein levels in MCF-7 mammospheres: (**A**) The representative image of the western blot results from 3 independent experiments; β-actin was used as an internal control. (**B**) The relative expression levels of PCNA (top) and ABCG2 (bottom) were analyzed by densitometry; β-actin was used as a normalization control (n = 3; *—*p* ≤ 0.05).

The ATP Binding Cassette Subfamily G Member 2 (ABCG2) functions as a transporter and plays a crucial role in the development of multi-drug resistance to chemotherapeutic agents [38]. ABCG2 has been shown to be a major chemotherapy resistance marker in MCF-7 cells [39]. Proliferating Cell Nuclear Antigen (PCNA) is a nuclear non-histone protein that is necessary for deoxyribonucleic acid (DNA) synthesis. PCNA is considered to be a marker of cell proliferation in various cancers. Upregulation of PCNA in breast cancers is associated with poor prognosis [40]. Therefore, in this study, we assessed the levels of ABCG2 and PCNA in MCF-7 mammospheres exposed to antibiotics (Figure 4A and Figure S3). ABCG2 was overexpressed in mammospheres treated with the antibiotics of the tetracycline class and chloramphenicol (Figure 4B). The PCNA level was reduced in chloramphenicol- and azithromycin-treated cells (Figure 4B).

## 3. Discussion

On the one hand, antibiotics have been shown to inhibit the population of CSCs in vitro; on the other hand, their effectiveness in clinical trials has produced inconsistent results [16]. We propose that this significant disparity may be associated with specific characteristics of the CSC microenvironment. In particular, hypoxia is a common feature of tumor growth, affecting a wide range of cellular processes [41]. The hypoxic microenvironment plays a critical role in regulating the self-renewal and metastatic potential of CSCs. Proteins belonging to the HIF family regulate the activity of transcription factors, including Octamer-binding transcription factor 4 (Oct4) and SRY-box transcription factor 2 (SOX2), which are involved in maintaining the stem cell phenotype and controlling self-renewal capacity [42,43]. Furthermore, SOX2 promotes the migration of breast cancer cells by inducing the expression of neural precursor cell expressed developmentally down-regulated protein 9 (NEDD9) [43]. NEDD9, in turn, promotes epithelial-mesenchymal transition (EMT) by upregulating the expression of snail family transcriptional repressor 1 (SNAI1, Snail) and snail family transcriptional repressor 2 (SNAI2, Slug) proteins [44]. Snail and Slug transcription factors inhibit the expression of E-cadherin, a calcium-dependent cell–cell adhesion molecule, thereby promoting EMT and changes in the actin cytoskeleton that facilitate cell motility [45,46]. Similar mechanisms have been demonstrated for several other HIF-regulated proteins.

In our study, we investigated the effects of five commonly used antibiotics (azithromycin, erythromycin, tetracycline, doxycycline, and chloramphenicol) on the mitochondrial biogenesis of breast cancer cells. Based on their biological activity, the tested compounds can be classified into two distinct groups: azithromycin, erythromycin, and chloramphenicol bind to the large subunit of the mitochondrial ribosome (39S), while tetracycline and doxycycline bind to the small subunit (28S) [16]. More than 60 mitochondria-related proteins are overexpressed in mammospheres [15], including those involved in electron transport (NDUFB10, COX6B1, PMPCA, COX5B, SDHA, UQCRC1), ATP synthesis (ATP5B, ATPIF1, ATP5A1, ATP5F1, ATP5H, ATP5O), and mitochondrial biogenesis (HSPA9, TIMM8A, GFM1, MRPL45, MRPL17, HSPD1, TSFM, TUFM), thereby making spheres are good models for the study.

Initially, we questioned if hypoxia affects the CSC number in the control culture. As we see in Figure 1C, the number of MCF-7 mammospheres was not affected by hypoxia. Similarly, the ratio of ALDH-bright cells remained unchanged under hypoxic conditions (Figure 2D). We also did not observe any differences in the percentage of ALDH-positive cells between anchorage-dependent and anchorage-independent culture conditions. Based on our results under chosen culture conditions, the lack of an effect of hypoxia on the proportion of CSC, measured by SFA and the ALDH-bright cell ratio count, let us conclude that all variations in CSC number detected further are related to the tested compound’s activity and do not reflect a direct impact of hypoxia on MCF-7 cells.

Furthermore, we assessed the impact of antibiotics on mitochondrial membrane potential, a well-established marker of overall mitochondrial metabolism. Among the five tested antibiotics, only doxycycline demonstrated a significant reduction in mitochondrial membrane potential compared to the control samples (Figure 3A,B). Therefore, we selected doxycycline for metabolic flux analysis of oxygen consumption rate, which revealed a substantial decrease in both maximal and basal respiration (Figure 3C,D). Consequently, doxycycline inhibits oxidative phosphorylation and impairs mitochondrial function in MCF-7 cell population. We did not observe such metabolic modulation activity with the other tested compounds.

The unresolved question was whether hypoxia could influence the eradication of CSCs by antibiotics. Our results indicate that chloramphenicol can inhibit mammosphere formation (Figure 1C) and reduce the ratio of ALDH-bright cells (Figure 2D) regardless of the oxygen levels in the culture. Doxycycline decreased the number of mammospheres regardless of the oxygen supply conditions and decreased the percentage of ALDH-bright cells in normoxia, but it did not lead to significant changes in the ALDH-bright cell content in hypoxia. Tetracycline inhibited sphere formation without affecting the ALDH-cell ratio in both normoxia and hypoxia. Azithromycin reduced the ALDH-bright cell ratio in both normoxia and hypoxia without inhibiting sphere formation. Erythromycin had no effect on mammosphere formation and showed a decrease in ALDH-bright cell percentage in normoxia without any effect in hypoxia.

The data obtained in conventional cell culture experiments once again highlighted the low convergence of results obtained between three-dimensional and two-dimensional culture conditions: erythromycin reduced the ALDH-bright cell ratio, while tetracycline and doxycycline increased it (Figure 2C). In the presence of azithromycin and chloramphenicol, we did not detect any differences in the ALDH-bright cell ratio, as compared to the control samples.

In summary, our findings demonstrate that chloramphenicol, doxycycline, and tetracycline effectively inhibit mammosphere formation under hypoxic conditions. Among these antibiotics, only chloramphenicol treatment resulted in a significant reduction in the ALDH-bright cell ratio when exposed to a 4% oxygen environment. Azithromycin reduced the ALDH-bright cell ratio in hypoxia without affecting sphere formation. Notably, chloramphenicol and doxycycline exhibited remarkable potential among all the antibiotics tested. Chloramphenicol consistently demonstrated the ability to target CSCs regardless of the oxygen conditions, while doxycycline showed potent targeting of CSCs and modulation of cellular metabolism.

Despite the ongoing debates surrounding the use of antibiotics in cancer therapy, numerous studies have been published over the past few decades highlighting the effectiveness of antibiotics in eradicating CSCs. According to published data, pre-operative treatment with oral doxycycline (200 mg per day) for two weeks led to a substantial reduction in CD44+ CSC number, ranging from 17.65% to 66.67%, in tumor samples from eight out of nine breast cancer patients (*p*-value < 0.005) [47]. Four patients exhibited reductions of 50% or more. Notably, the observed reductions in CD44+ CSCs induced by doxycycline were independent of histological grade (1, 2, 3), tumor diameter (small, large), and molecular subtype.

In another study, azithromycin enhanced favorable results of chemotherapy in patients with advanced non-small-cell lung cancer (stage III–IV): one-year survival rates increased from 45.0% to 75.0%. There was also an improvement in the median survival time from 12.0 to 13.0 months [19].

An advantage of antibiotic use in combined therapies for breast cancer is that they have already received approval by the food and drug administration and can be safely used in long-term treatments. The mechanism of the action of antibiotics is associated with the suppression of mitochondrial biogenesis by disrupting protein synthesis in cancer cell mitochondria [16].

Metastatic dissemination and drug resistance are the leading causes of high mortality rates in breast cancer patients [48]. Conventional therapies primarily target rapidly dividing bulk tumor cells, while dormant cancer cells, including CSCs, remain unaffected and can lead to fatal recurrences years and sometimes even decades later [49]. One of the strategies to improve the overall survival rate and to prevent cancer recurrence is to develop combination therapies that target not only the bulk tumor, but also the small yet highly aggressive population of CSCs. R. Lamb, with colleagues, demonstrated that azithromycin, doxycycline, tigecycline, and chloramphenicol could effectively target CSC populations across eight different tumor types under normoxic conditions [16]. Another study by E. Gottlieb’s group showed that combination treatment with imatinib and tigecycline selectively eradicated leukemic stem cells in vitro and in vivo using a xenotransplantation model of human chronic myeloid leukemia cells [50]. Additionally, the anti-malarial drug atovaquone, a potent and selective inhibitor of oxidative phosphorylation (OXPHOS), induced apoptosis of ALDH-positive CSCs and inhibited mammosphere formation in sphere culture [51]. The prominent feature of CSCs is their ability to self-renew without losing their proliferative capacity with each cell division [52]. CSCs exhibit higher proliferation and growth potential, along with elevated expression of resistance markers, compared to the bulk population [53]. Ki-67 and PCNA are commonly used as measures of tumor cell proliferation. Breast cancer tumors with high Ki-67 (>20%) have been associated with a higher probability of disease relapse (79.1% versus 55.3%) and death (95.6% versus 71%) within four years, when compared to tumors with low Ki-67 values [54]. Breast cancers with high PCNA scores (≥25) have been associated with shorter disease-free (*p* = 0.007) and overall survival (*p* = 0.01) times [40].

In our study, we observed a decrease in PCNA protein levels in mammospheres treated with azithromycin and chloramphenicol, compared to the control samples. Both of these compounds also reduced the ALDH-bright cell ratio in mammospheres under normoxic and hypoxic conditions. Moreover, chloramphenicol inhibited mammosphere formation regardless of oxygen levels. Therefore, we suggest that among the tested antibiotics, azithromycin and chloramphenicol could be considered as potential candidates for combined therapy in tumors with high cell proliferation indices. Importantly, these two antibiotics exhibit lower toxicity compared to the cytostatic drug docetaxel, and could therefore be used for long-term therapy, without serious side effects, for cancer-cell targeting [55].

Drug resistance represents a significant challenge in breast cancer therapy, leading to reduced survival among patients [56]. One key player in the development of resistance is the ATP-binding cassette (ABC) transporter family member known as ABCG2, or breast cancer resistance protein (BCRP) [57]. ABCG2 extrudes various compounds, including therapeutic agents and other endogenous or exogenous toxic substances, from cancer cells, contributing to multidrug resistance (MDR) [38]. Furthermore, ABCG2 has been identified as a potential marker for stem cells and a novel target for cancer therapy [38,42]. However, the use of antibiotics is also complicated by the emergence of resistance. The possible influence on the rate and speed of MDR development might be the key factor against the use of antibiotic therapy in cancer patients. Our findings revealed an increase in ABCG2 protein levels in spheres treated with tetracycline, doxycycline, or chloramphenicol. Conversely, we did not observe upregulation of ABCG2 in mammospheres treated with azithromycin and erythromycin. Interestingly, azithromycin reduced the ALDH-bright cell ratio in mammospheres under both normoxic and hypoxic conditions without affecting the number of sphere-forming cells. These results suggest that the use of chloramphenicol and doxycycline in treatment-naïve patients may influence MDR development. Further research has to be conducted in order to determine the optimal implementation time for the antibiotics in a BC treatment scheme. Additionally, azithromycin may have potential early-stage applications to decrease the ALDH-bright cell ratio in the bloodstream in order to control the spread of disease.

## 4. Materials and Methods

### 4.1. Cell Culture

Breast cancer epithelial cell line MCF-7 (ATCC number: HTB-22) was kindly provided by the laboratory of Prof. Jenny L. Persson (Umea University, Sweden). Human skin fibroblasts (HSF) were established and kindly provided by Dr. Elena Zakirova (Kazan Federal University, Russia). MCF-7 and HSF cells were cultured with Roswell Park Memorial Institute (RPMI)-1640 medium (PanEco, Moscow, Russia) containing 10% fetal bovine serum (Gibco, Thermo Fisher Scientific, MA, USA) and 10% penicillin-streptomycin (PanEco, Moscow, Russia) in a 5% CO_2_ incubator (Eppendorf, Hamburg, Germany) at 37 °C.

### 4.2. Sphere Formation Assay

The sphere culture medium (SCM) was composed of Dulbecco‘s Modified Eagle Medium/F12 (DMEM/F12) (PanEco, Moscow, Russia) media supplemented by B27 (final concentration 1×, PanEco, Russia), 40 ng/mL epidermal growth factor (EGF), and 40 ng/mL fibroblast growth factor 2 (FGF2) (SCI store, Moscow, Russia). Cells at the subconfluent level were trypsinized and cell pellets were resuspended in SCM at a concentration of 10^6^ cells/mL. Then, 100 µL of cell suspension was transferred into a tube, containing 900 µL of SCM. Cells were gently pipetted several times through a needle of a 1.0 mL sterile insulin syringe to disintegrate cell clots. Cell number was determined in the Bürker counting chamber (Thermo Fisher Scientific, MA, USA). A total of 4000 cells/dish were seeded and cultured in non-adherent 35 × 10 mm culture dishes (SPL Life Sciences Co., Pocheon-si, Republic of Korea) containing SCM with one of five antibiotics (Sigma-Aldrich, MO, USA) or the chemotherapeutic drug docetaxel (0.5 nM) (Sandoz, Holzkirchen, Germany). Cells were grown for two weeks in a humidified incubator in 5% carbon dioxide and 21% (normoxic) or 4% (hypoxic) oxygen supply conditions. Hypoxic conditions were created in a custom-made BACTROX hypoxic chamber (Sheldon Manufacturing, Inc., OR, USA). Spheres were counted and photomicrographs were captured under a Leica DM IL Led Fluo microscope (Leica Microsystems, Wetzlar, Germany) equipped with a Leica DFC365 FX camera. All the experiments were performed in technical duplicates with five independent experiments.

### 4.3. Western-Blot Analysis

Mammospheres were obtained by culturing the dissociated MCF-7 cells for 2 weeks in SCM, as described in the Section 4.2 Sphere Formation Assay. Spheres were lysed in a Radioimmunoprecipitation assay buffer (RIPA-buffer) containing protease inhibitors (PI) (Thermo Fisher Scientific, MA, USA) and phenylmethylsulfonyl fluoride (PMSF (Thermo Fisher Scientific, MA, USA) for 40 min on ice and centrifuged. Protein concentrations in lysates were calculated using a bicinchoninic acid (BCA) assay (Thermo Fisher Scientific, MA, USA). Proteins were separated by molecular weight in polyacrylamide gel electrophoresis with 4% stacking and 8% separating gels, and 30 µg of a protein sample mixed with 4× loading buffer was loaded into each well of gel. After electrophoresis, proteins were electroblotted in semi-dry transfer from gel to polyvinylidene difluoride (PVDF) membrane (Bio-Rad Laboratories, CA, USA) according to BioRad standard protocol. Transfer accuracy was checked by gel staining with Ponceau S (Sigma-Aldrich, USA). Blocking of non-specific binding was achieved by placing the membrane overnight in a 5% solution of dry milk in a tris-buffered saline with tween-20 (TBST-buffer). Later, for protein detection, membranes were incubated in a solution of primary antibodies, diluted with a ratio of 1:200: ABCG2 (cat. no. sc-58222; Santa Cruz Biotechnology, TX, USA), PCNA (cat. no. sc-7909; Santa Cruz Biotechnology, USA), and β-actin (cat. no. A00730; GenScript Biotech, NJ, USA). Further membranes were exposed to horseradish peroxidase (HRP)-conjugated secondary antibodies (Anti-rabbit IgG cat. no. A16110 or Anti-mouse IgG cat. no. A16078; Thermo Fisher Scientific, USA). Visualization of labeled proteins of interest was achieved after placing the membranes in BioRad HRP-substrate solution for several minutes.

### 4.4. Cell Proliferation Assay

Exponentially growing MCF-7 cells were trypsinized, stained with trypan blue, counted, and seeded in a 96-well plate (2000 cells per well). Upon overnight incubation, antibiotics or Docetaxel (Taxotere, Aventis Pharma SA, Croissy-Beaubourg, France) as a positive control compound were added to the cells and incubated for 72 h. For antibiotics, we used the following concentrations range: 1.85–450 µM azithromycin, 1.85–450 µM erythromycin, 9.26 µM–2.25 mM chloramphenicol, 930 nM–225 µM doxycycline, and 1.85–450 µM tetracycline. 3-(4,5-dimethylthiazol-2-yl)-2,5-diphenyltetrazolium bromide (MTT) assay (PanEco, Moscow, Russia) was performed according to manufacturer’s protocol. Absorbance was measured at 590 nM on the Tecan Infinite M200 Pro multimode plate reader (Tecan Group Ltd., Männedorf, Switzerland). Cell culture media background was subtracted during the analysis.

### 4.5. Mitochondrial Membrane Potential Measurement

We used antibiotics to target CSCs mitochondria as a mild side-effect and more than 60 mitochondrial proteins were reported to be overexpressed in MCF-7 mammospheres when compared with monolayers [15]. Therefore, we wanted to test the influence of antibiotics on mitochondria. For this purpose, we stained MCF-7 and HSF cells with JC-1 carbocationic dye—a sensor of mitochondrial transmembrane potential. This dye exhibits potential-dependent accumulation in mitochondria. When mitochondrial membrane potential is high, JC-1 accumulation causes a fluorescence emission shift from green to red due to a concentration-dependent formation of red fluorescent aggregates.

MCF-7 or HSF cells were plated at 30,000 cells/cm^2^ and cultured for 48 h in the presence of 1 out of 5 antibiotics: 50 µM azithromycin, 50 µM erythromycin, 250 µM chloramphenicol, 25 µM doxycycline, or 50 µM tetracycline. Cells then were washed with phosphate-buffered saline (PBS) and stained for 20 min with 8 µM JC-1 dye (Thermo Fisher Scientific, MA, USA) in PBS. Further, cells were washed 3 times with RPMI-1640 culture media supplemented with 10% fetal bovine serum (FBS) and incubated for 24 h in culture media with antibiotics. Cells were captured on a Carl Zeiss Axio Observer Z.1 microscope (Zeiss AG, Oberkochen, Germany) equipped with an AxioCam MRc5 camera and HXP 120 C lightning unit. In polarized mitochondria, JC-1 dye forms aggregates that result in red fluorescence; in cells with depolarized mitochondrial membrane, monomers emit green fluorescence.

### 4.6. Evaluation of Mitochondrial Function

Real-time OCR for MCF-7 cells were determined using the Seahorse Extracellular Flux analyzer (Seahorse Bioscience, MA, USA). The Cell Mito Stress Test is a common assay to measure the OCR of live cells for the evaluation of mitochondrial respiration function: basal, maximal, non-mitochondrial respiration, and proton leak. Briefly, 20,000 of MCF-7 cells per well were seeded into XFe-96-well cell culture plates. Cells were treated with 25 µM doxycycline or vehicle control for 24 h. Then, cells were washed 3 times in a pre-warmed XF assay media supplemented with 10 mM glucose, 1 mM Pyruvate, and 2 mM L-glutamine and were adjusted at 7.4 pH (Seahorse Bioscience, MA, USA). Cells were then maintained in 180 μL/well of XF assay media at 37 °C, in a non-CO_2_ incubator for 1 h to degas before running the assay. During the incubation time, 20 μL of 20 μM oligomycin, 22 μL of 10 μM FCCP, 25 μL of 5 μM rotenone and 5 μM antimycin mix (Seahorse Bioscience, MA, USA) were loaded in XF assay media into the injection ports in the XFe-96 sensor cartridge. We used Hoechst 33,342 (Thermo Fischer Scientific, MA, USA) staining to measure cell density for data normalization. The fluorescence measurement was performed using the Infinite M200 Pro plate reader (Tecan Group Ltd., Zurich, Switzerland). Data set was analyzed by Seahorse Wave Desktop software version 2.6.1 (Seahorse Bioscience, MA, USA).

### 4.7. ALDEFLUOR Assay

Mammospheres were obtained by culturing the dissociated MCF-7 cells for 2 weeks in SCM, as described in Section 4.2 (Sphere Formation Assay). For measuring ALDH-activity in mammospheres, spheres were gently trypsinized and pipetted to obtain single cell suspension. MCF-cells cultured in the presence of antibiotics and mammosphere-derived cells were stained with the ALDEFLUOR reagent (StemCell Technologies, Vancouver, BC, Canada), according to the manufacturers protocol. Briefly, the total number of 1 × 10^6^ cells were re-suspended in 1 mL assay buffer; further 6 μL of ALDEFLUOR reagent was added, and 500 µL of this cell suspension was transferred into a new tube with 6 μL of diethylaminobenzaldehyde (DEAB) regent. Cells were incubated for 30 min at 37 °C, centrifuged, and resuspended in 500 μL of Assay Buffer and subjected to an analysis on a FACSAria III flow cytometer (Becton Dickinson, NJ, USA).

### 4.8. Statistical Analysis

In vitro data are represented as the mean ± standard error of the mean (SEM), taken over ≥3 independent experiments, with ≥2 technical replicates per each experiment, unless specified otherwise. *p* ≤ 0.05 was considered significant. Statistical significance was measured using the nonparametric Mann–Whitney U test.

## 5. Conclusions

Repurposing FDA-approved antibiotics that exert suppressive effects on CSCs by the “off-target” inhibition of mitochondrial biogenesis has emerged as a strategy for CSC targeting [16,47]. In our study, we assessed the influence of five antibiotics on CSC populations under both normoxic and hypoxic conditions. Our findings indicate that the hypoxic microenvironment does not significantly alter the number of breast CSCs, but the inhibitory effects of antibiotics on CSCs may be attenuated under low-oxygen conditions. Chloramphenicol demonstrated a condition-independent ability to inhibit CSCs, whereas doxycycline exhibited the capacity to modulate overall cellular metabolism and inhibit sphere formation under both normoxic and hypoxic conditions, albeit without reducing the proportion of ALDH-bright cells in hypoxia. Notably, the major challenge for chloramphenicol and doxycycline is associated with the development of multidrug resistance through the upregulation of drug efflux transport systems.

Conversely, treatment with azithromycin significantly suppressed cell proliferation and reduced the ratio of ALDH-bright cells in mammospheres. Importantly, azithromycin did not induce upregulation of ABCG2, a key contributor to multidrug resistance. However, it failed to inhibit mammosphere formation in hypoxia. Based on our findings, azithromycin may be a preferred compound for early-stage disease progression, while the introduction of chloramphenicol and doxycycline may be preferred in late-line treatment regimens due to a higher risk of promoting multidrug resistance in breast cancer cells.

## Figures and Tables

**Table 1:** The list of tested antibiotics, concentrations, and molecular targets.

Antibiotic	Target	Tested Concentration	Concentration in This Study
Azithromycin	The 39S large mitoribosomal subunit	1.85 µM–450 µM	50 µM
Erythromycin	The 39S large mitoribosomal subunit	1.85 µM–450 µM	50 µM
Chloramphenicol	The 39S large mitoribosomal subunit	9.26 µM–2.25 mM	250 µM
Doxycycline	The 28S small mitoribosomal subunit	930 nM–225 µM	25 µM
Tetracycline	The 28S small mitoribosomal subunit	1.85 µM–450 µM	50 µM

## Supplementary Materials

The following supporting information can be downloaded at: https://www.mdpi.com/article/10.3390/ijms241411540/s1.

## References

[1] Clarke M.F., Dick J.E., Dirks P.B., Eaves C.J., Jamieson C.H.M., Jones D.L., Visvader J., Weissman I.L., Wahl G.M. (2006). Cancer Stem Cells—Perspectives on Current Status and Future Directions: AACR Workshop on Cancer Stem Cells. Cancer Res..

[2] Di Tomaso T., Mazzoleni S., Wang E., Sovena G., Clavenna D., Franzin A., Mortini P., Ferrone S., Doglioni C., Marincola F.M. (2010). Immunobiological Characterization of Cancer Stem Cells Isolated from Glioblastoma Patients. Clin. Cancer Res..

[3] Tirino V., Desiderio V., Paino F., De Rosa A., Papaccio F., La Noce M., Laino L., De Francesco F., Papaccio G. (2013). Cancer stem cells in solid tumors: An overview and new approaches for their isolation and characterization. FASEB J..

[4] Zhou W., Choi M., Margineantu D., Margaretha L., Hesson J., Cavanaugh C., Blau C.A., Horwitz M.S., Hockenbery D., Ware C. (2012). HIF1α induced switch from bivalent to exclusively glycolytic metabolism during ESC-to-EpiSC/hESC transition. EMBO J..

[5] Prigione A., Fauler B., Lurz R., Lehrach H., Adjaye J. (2010). The senescence-related mitochondrial/oxidative stress pathway is repressed in human induced pluripotent stem cells. Stem Cells.

[6] Liu P.P., Liao J., Tang Z.J., Wu W.J., Yang J., Zeng Z.L., Hu Y., Wang P., Ju H.Q., Xu R.H. (2014). Metabolic regulation of cancer cell side population by glucose through activation of the Akt pathway. Cell Death Differ..

[7] Ciavardelli D., Rossi C., Barcaroli D., Volpe S., Consalvo A., Zucchelli M., De Cola A., Scavo E., Carollo R., D’Agostino D. (2014). Breast cancer stem cells rely on fermentative glycolysis and are sensitive to 2-deoxyglucose treatment. Cell Death Dis..

[8] Liao J., Qian F., Tchabo N., Mhawech-Fauceglia P., Beck A., Qian Z., Wang X., Huss W.J., Lele S.B., Morrison C.D. (2014). Ovarian Cancer Spheroid Cells with Stem Cell-Like Properties Contribute to Tumor Generation, Metastasis and Chemotherapy Resistance through Hypoxia-Resistant Metabolism. PLoS ONE.

[9] De Luca A., Fiorillo M., Peiris-Pagès M., Ozsvari B., Smith D.L., Sanchez-Alvarez R., Martinez-Outschoorn U.E., Cappello A.R., Pezzi V., Lisanti M.P. (2015). Mitochondrial biogenesis is required for the anchorage-independent survival and propagation of stem-like cancer cells. Oncotarget.

[10] Grazia Cipolleschi M., Marzi I., Santini R., Fredducci D., Cristina Vinci M., D’Amico M., Rovida E., Stivarou T., Torre E., Dello Sbarba P. (2014). Hypoxia-resistant profile implies vulnerability of cancer stem cells to physiological agents, which suggests new therapeutic targets. Cell Cycle.

[11] Fiorillo M., Sotgia F., Lisanti M.P. (2019). “Energetic” Cancer Stem Cells (e-CSCs): A New Hyper-Metabolic and Proliferative Tumor Cell Phenotype, Driven by Mitochondrial Energy. Front. Oncol..

[12] Koc E.C., Burkhart W., Blackburn K., Moyer M.B., Schlatzer D.M., Moseley A., Spremulli L.L. (2001). The large subunit of the mammalian mitochondrial ribosome. Analysis of the complement of ribosomal proteins present. J. Biol. Chem..

[13] Stefano G.B., Samuel J., Kream R.M. (2017). Antibiotics May Trigger Mitochondrial Dysfunction Inducing Psychiatric Disorders. Med. Sci. Monit..

[14] Ferreri A.J., Ponzoni M., Guidoboni M., Resti A.G., Politi L.S., Cortelazzo S., Demeter J., Zallio F., Palmas A., Muti G. (2006). Bacteria-eradicating therapy with doxycycline in ocular adnexal MALT lymphoma: A multicenter prospective trial. J. Natl. Cancer Inst..

[15] Lamb R., Harrison H., Hulit J., Smith D.L., Lisanti M.P., Sotgia F. (2014). Mitochondria as new therapeutic targets for eradicating cancer stem cells: Quantitative proteomics and functional validation via MCT1/2 inhibition. Oncotarget.

[16] Lamb R., Ozsvari B., Lisanti C.L., Tanowitz H.B., Howell A., Martinez-Outschoorn U.E., Sotgia F., Lisanti M.P. (2015). Antibiotics that target mitochondria effectively eradicate cancer stem cells, across multiple tumor types: Treating cancer like an infectious disease. Oncotarget.

[17] Han J.J., Kim T.M., Jeon Y.K., Kim M.K., Khwarg S.I., Kim C.W., Kim I.H., Heo D.S. (2015). Long-term outcomes of first-line treatment with doxycycline in patients with previously untreated ocular adnexal marginal zone B cell lymphoma. Ann. Hematol..

[18] Ferreri A.J., Ponzoni M., Guidoboni M., De Conciliis C., Resti A.G., Mazzi B., Lettini A.A., Demeter J., Dell’Oro S., Doglioni C. (2005). Regression of ocular adnexal lymphoma after Chlamydia psittaci-eradicating antibiotic therapy. J. Clin. Oncol..

[19] Chu D.J., Yao D.E., Zhuang Y.F., Hong Y., Zhu X.C., Fang Z.R., Yu J., Yu Z.Y. (2014). Azithromycin enhances the favorable results of paclitaxel and cisplatin in patients with advanced non-small cell lung cancer. Genet. Mol. Res..

[20] Harris A.L. (2002). Hypoxia—A key regulatory factor in tumour growth. Nat. Rev. Cancer.

[21] Ullah A., Leong S.W., Wang J., Wu Q., Ghauri M.A., Sarwar A., Su Q., Zhang Y. (2021). Cephalomannine inhibits hypoxia-induced cellular function via the suppression of APEX1/HIF-1α interaction in lung cancer. Cell Death Dis..

[22] Liao D., Johnson R.S. (2007). Hypoxia: A key regulator of angiogenesis in cancer. Cancer Metastasis Rev..

[23] Ullah A., Ullah N., Nawaz T., Aziz T. (2023). Molecular Mechanisms of Sanguinarine in Cancer Prevention and Treatment. Anti-Cancer Agents Med. Chem..

[24] Ginouvès A., Ilc K., Macías N., Pouysségur J., Berra E. (2008). PHDs overactivation during chronic hypoxia “desensitizes” HIFα and protects cells from necrosis. Proc. Natl. Acad. Sci. USA.

[25] Semenza G.L. (2003). Targeting HIF-1 for cancer therapy. Nat. Rev. Cancer.

[26] Vaupel P., Mayer A. (2007). Hypoxia in cancer: Significance and impact on clinical outcome. Cancer Metastasis Rev..

[27] D’Alterio C., Scala S., Sozzi G., Roz L., Bertolini G. (2020). Paradoxical effects of chemotherapy on tumor relapse and metastasis promotion. Semin. Cancer Biol..

[28] Karagiannis G.S., Condeelis J.S., Oktay M.H. (2018). Chemotherapy-induced metastasis: Mechanisms and translational opportunities. Clin. Exp. Metastasis.

[29] Duivenvoorden W.C., Hirte H.W., Singh G. (1997). Use of tetracycline as an inhibitor of matrix metalloproteinase activity secreted by human bone-metastasizing cancer cells. Invasion Metastasis.

[30] Van den Bogert C., van Kernebeek G., de Leij L., Kroon A.M. (1986). Inhibition of mitochondrial protein synthesis leads to proliferation arrest in the G1-phase of the cell cycle. Cancer Lett..

[31] Iwasaki H., Inoue H., Mitsuke Y., Badran A., Ikegaya S., Ueda T. (2002). Doxycycline induces apoptosis by way of caspase-3 activation with inhibition of matrix metalloproteinase in human T-lymphoblastic leukemia CCRF-CEM cells. J. Lab. Clin. Med..

[32] Zhang M., Gao C.E., Chen W.L., Tang Y.Y., Nie J.Y., Shen L.D., Ma X., Chen D.D. (2018). Opposite response to hypoxia by breast cancer cells between cell proliferation and cell migration: A clue from microRNA expression profile. Oncol. Lett..

[33] Hubbi M.E., Semenza G.L. (2015). Regulation of cell proliferation by hypoxia-inducible factors. Am. J. Physiol. Cell Physiol..

[34] Ciccone V., Terzuoli E., Donnini S., Giachetti A., Morbidelli L., Ziche M. (2018). Stemness marker ALDH1A1 promotes tumor angiogenesis via retinoic acid/HIF-1alpha/VEGF signalling in MCF-7 breast cancer cells. J. Exp. Clin. Cancer Res..

[35] Vassalli G. (2019). Aldehyde Dehydrogenases: Not Just Markers, but Functional Regulators of Stem Cells. Stem Cells Int..

[36] Lin C.C., Lo M.C., Moody R.R., Stevers N.O., Tinsley S.L., Sun D. (2018). Doxycycline targets aldehyde dehydrogenase-positive breast cancer stem cells. Oncol. Rep..

[37] Li G.B., Fu R.Q., Shen H.M., Zhou J., Hu X.Y., Liu Y.X., Li Y.N., Zhang H.W., Liu X., Zhang Y.H. (2017). Polyphyllin I induces mitophagic and apoptotic cell death in human breast cancer cells by increasing mitochondrial PINK1 levels. Oncotarget.

[38] Mo W., Zhang J.T. (2012). Human ABCG2: Structure, function, and its role in multidrug resistance. Int. J. Biochem. Mol. Biol..

[39] Doyle L.A., Yang W., Abruzzo L.V., Krogmann T., Gao Y., Rishi A.K., Ross D.D. (1998). A multidrug resistance transporter from human MCF-7 breast cancer cells. Proc. Natl. Acad. Sci. USA.

[40] Tahan S.R., Neuberg D.S., Dieffenbach A., Yacoub L. (1993). Prediction of early relapse and shortened survival in patients with breast cancer by proliferating cell nuclear antigen score. Cancer.

[41] Folkman J., Shing Y. (1992). Angiogenesis. J. Biol. Chem..

[42] Ding X.W., Wu J.H., Jiang C.P. (2010). ABCG2: A potential marker of stem cells and novel target in stem cell and cancer therapy. Life Sci..

[43] Wang Y., Bibi M., Min P., Deng W., Zhang Y., Du J. (2019). SOX2 promotes hypoxia-induced breast cancer cell migration by inducing NEDD9 expression and subsequent activation of Rac1/HIF-1α signaling. Cell. Mol. Biol. Lett..

[44] Kong C., Wang C., Wang L., Ma M., Niu C., Sun X., Du J., Dong Z., Zhu S., Lu J. (2011). NEDD9 is a positive regulator of epithelial-mesenchymal transition and promotes invasion in aggressive breast cancer. PLoS ONE.

[45] Russell H., Pranjol M.Z.I. (2018). Transcription factors controlling E-cadherin down-regulation in ovarian cancer. Biosci. Horiz..

[46] Leggett S.E., Hruska A.M., Guo M., Wong I.Y. (2021). The epithelial-mesenchymal transition and the cytoskeleton in bioengineered systems. Cell Commun. Signal..

[47] Scatena C., Roncella M., Di Paolo A., Aretini P., Menicagli M., Fanelli G., Marini C., Mazzanti C.M., Ghilli M., Sotgia F. (2018). Doxycycline, an Inhibitor of Mitochondrial Biogenesis, Effectively Reduces Cancer Stem Cells (CSCs) in Early Breast Cancer Patients: A Clinical Pilot Study. Front. Oncol..

[48] Eccles S.A., Aboagye E.O., Ali S., Anderson A.S., Armes J., Berditchevski F., Blaydes J.P., Brennan K., Brown N.J., Bryant H.E. (2013). Critical research gaps and translational priorities for the successful prevention and treatment of breast cancer. Breast Cancer Res..

[49] Goss P.E., Chambers A.F. (2010). Does tumour dormancy offer a therapeutic target?. Nat. Rev. Cancer.

[50] Kuntz E.M., Baquero P., Michie A.M., Dunn K., Tardito S., Holyoake T.L., Helgason G.V., Gottlieb E. (2017). Targeting mitochondrial oxidative phosphorylation eradicates therapy-resistant chronic myeloid leukemia stem cells. Nat. Med..

[51] Fiorillo M., Lamb R., Tanowitz H.B., Mutti L., Krstic-Demonacos M., Cappello A.R., Martinez-Outschoorn U.E., Sotgia F., Lisanti M.P. (2016). Repurposing atovaquone: Targeting mitochondrial complex III and OXPHOS to eradicate cancer stem cells. Oncotarget.

[52] Soltysova A., Altanerova V., Altaner C. (2005). Cancer stem cells. Neoplasma.

[53] Alowaidi F., Hashimi S.M., Alqurashi N., Alhulais R., Ivanovski S., Bellette B., Meedenyia A., Lam A., Wood S. (2018). Assessing stemness and proliferation properties of the newly established colon cancer ‘stem’ cell line, CSC480 and novel approaches to identify dormant cancer cells. Oncol. Rep..

[54] Veronese S.M., Gambacorta M., Gottardi O., Scanzi F., Ferrari M., Lampertico P. (1993). Proliferation index as a prognostic marker in breast cancer. Cancer.

[55] Sloan B., Scheinfeld N. (2008). The use and safety of doxycycline hyclate and other second-generation tetracyclines. Expert Opin. Drug Saf..

[56] Chong K.-H., Chang Y.-J., Hsu W.-H., Tu Y.-T., Chen Y.-R., Lee M.-C., Tsai K.-W. (2022). Breast Cancer with Increased Drug Resistance, Invasion Ability, and Cancer Stem Cell Properties through Metabolism Reprogramming. Int. J. Mol. Sci..

[57] Leimanis M.L., Georges E. (2007). ABCG2 membrane transporter in mature human erythrocytes is exclusively homodimer. Biochem. Biophys. Res. Commun..

