# Supplementary material for: Unravelling the Therapeutic Potential of Antibiotics in Hypoxia in a Breast Cancer MCF-7 Cell Line Model
Source: Int J Mol Sci. 2023 Jul 16;24(14):11540. doi: 10.3390/ijms241411540 (PMC10380719; doi:10.3390/ijms241411540)
Supplement: Supplementary file 1 [file ijms-24-11540-s001.zip › ijms-2472115-supplementary.pdf]

Supplementary Material

1 Supplementary Figures and Tables

1.1 Supplementary Figures

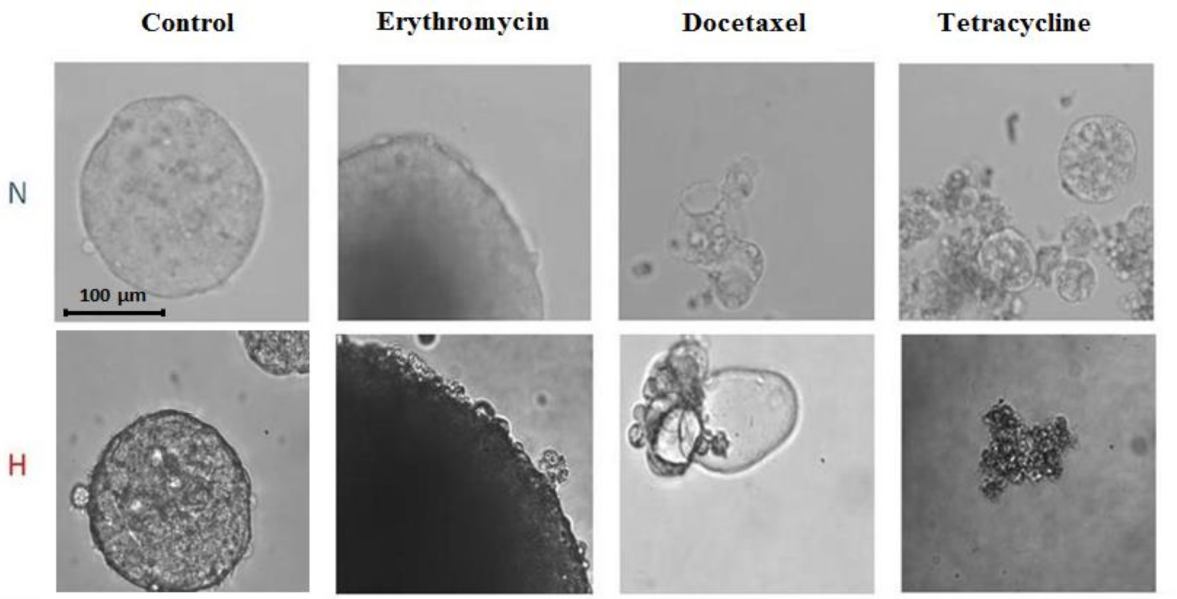

Supplementary Figure S1. Changes in morphology, caused by antibiotics in: **N** – normoxia, **H** –hypoxia.

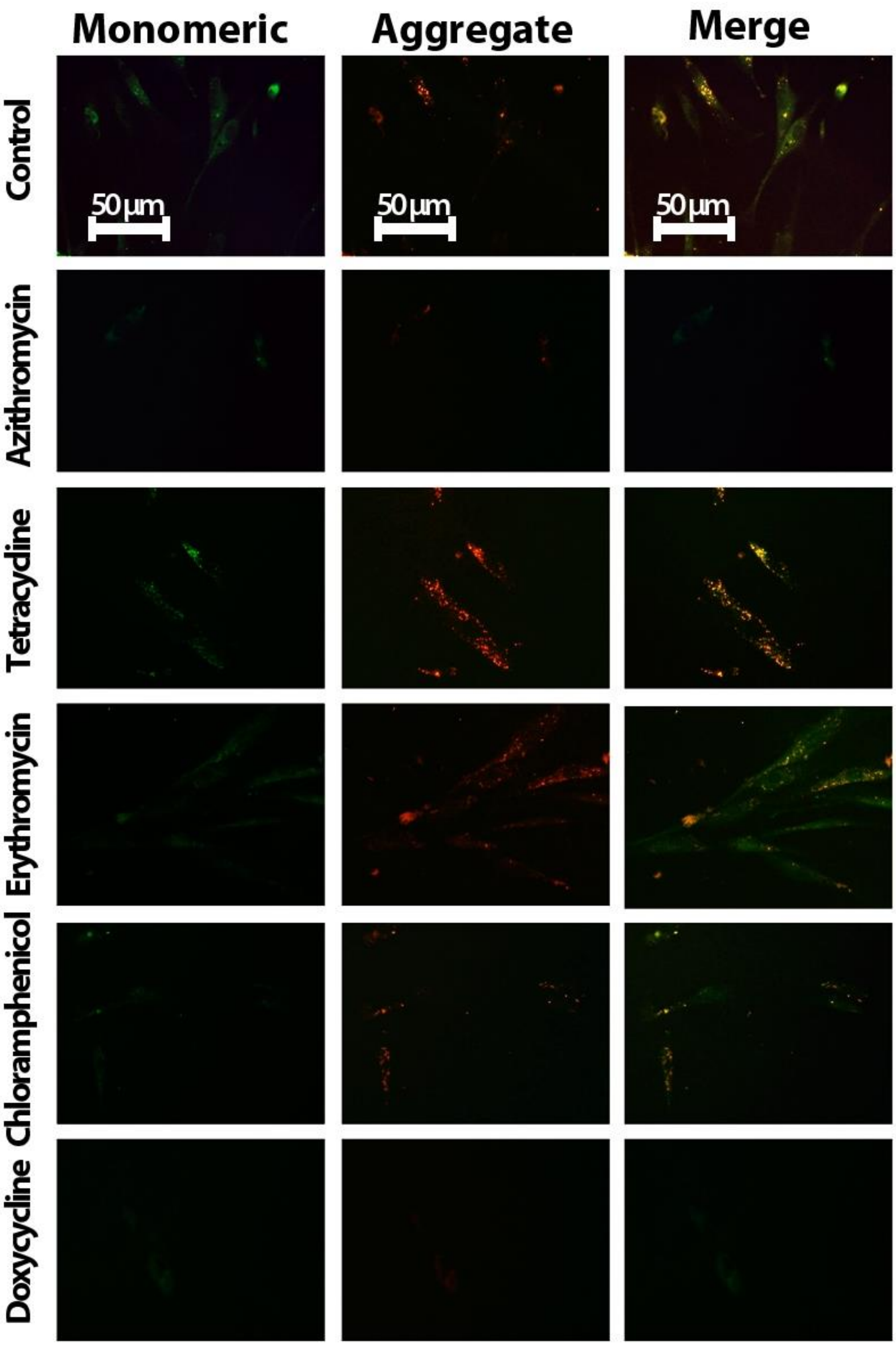

Supplementary Figure S2. JC-1 staining of mitochondrial network in Human Skin Fibroblasts after exposure to antibiotics; shift from red to green fluorescence represents depolarization of mitochondrial membrane potential; scale bar = 50 µm.

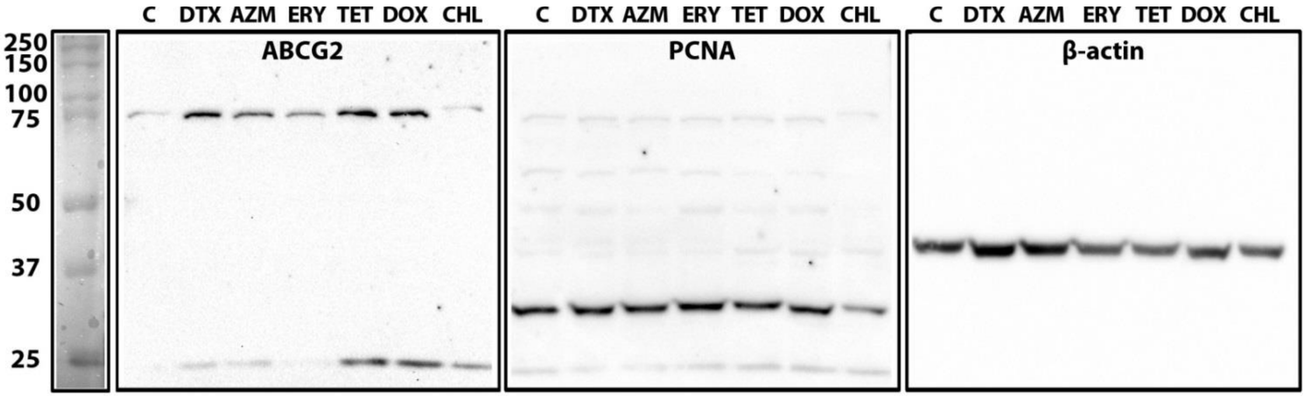

Supplementary Figure S3. Mammospheres cell lysates were analyzed by western blot using antibodies against **ABCG2**, **PCNA** and **β-actin**. Representative whole blots of Figure 4A are shown: **C** – control sample, **DTX** – docetaxel, **AZM** – azithromycin, **ERY** – erythromycin, **TET** – tertacycline, **DOX** – doxycycline, **CHL** – chloramphenicol.
